# Supplementary material for: Complexome profiling of the Chlamydomonas psb28 mutant reveals TEF5 as an early PSII assembly factor
Source: Plant Cell. 2025 Mar 17;37(6):koaf055. doi: 10.1093/plcell/koaf055 (PMC12164590; doi:10.1093/plcell/koaf055)
Supplement: koaf055_Supplementary_Data [file koaf055_supplementary_data.zip › psb28_interactive.zip!/plots/intensity/qualityPlots/peptides/ASA5_Cre17.g721300.t1.2.html]

### Cre17.g721300.t1.2

Trivial name: ASA5  
SeqCoverage [median]: 54.50  
Mass: 14.32 kDa  
  
 

| Sequence | MissedCleavage | Mass [Da] | wtIntensity | muIntensity | Score |
| --- | --- | --- | --- | --- | --- |
| FNYAYDR | 0 | 947.41 | 13354.22 | 16794.38 | 137.18 |
| FNYAYDRDLR | 1 | 1331.63 | 28254.68 | 42447.57 | 136.96 |
| LLIWQTQPSLQ | 0 | 1325.73 | 4385.06 | 0.00 | 192.26 |
| MLAANNKVYEAFNPSSK | 1 | 1882.92 | 826.80 | 0.00 | 69.73 |
| MLAANNKVYEAFNPSSKR | 2 | 2039.03 | 0.00 | 817.40 | 57.60 |
| NQVLESLHHTPAK | 0 | 1472.77 | 44585.52 | 65532.42 | 139.30 |
| RLLIWQTQPSLQ | 1 | 1481.84 | 2354.69 | 0.00 | 96.89 |
| SVSDHVMK | 0 | 901.43 | 16589.94 | 16963.73 | 130.27 |
| VYEAFNPSSK | 0 | 1140.55 | 42377.43 | 56384.17 | 167.12 |
| VYEAFNPSSKR | 1 | 1296.65 | 11034.02 | 17609.61 | 105.20 |
| YHETIWK | 0 | 975.48 | 4265.90 | 5661.44 | 128.48 |
|  |  |  | 168028.3 | 222210.7 |  |
